# The Role of Theatre Testing in Prevention Science

**DOI:** 10.1007/s10935-025-00832-3

**Published:** 2025-03-13

**Authors:** Georgina Warner, Anna Pérez-Aronsson

**Affiliations:** 1 Child Health and Parenting (CHAP), Department of Public Health and Caring Sciences, Uppsala University, Husargatan 3, Uppsala, 751 23 Sweden; 2 Centre for Women’s Mental Health During the Reproductive Lifespan– Womher, Uppsala University, Uppsala, Sweden

**Keywords:** Theatre testing, Implementation, Intervention, Prevention, Social media, Gender-based violence

## Abstract

Theatre Testing, a method extensively employed in marketing research and advertising, involves presenting materials to an audience in a controlled setting to gather feedback and insights. While the application of Theatre Testing in marketing and advertising is long-standing, its increasing application in prevention science raises several critical questions. This article describes the Theatre Testing process, debates the merits and drawbacks of the method, and exemplifies the pros and cons by discussing the method in relation to gender-based violence prevention within the Swedish ‘open preschool’ context. Advocates of Theatre Testing argue that simulating interventions before actual implementation offers valuable insights into participant responses, which can enhance the acceptability and effectiveness of these interventions. However, the controlled setting of Theatre Testing may not accurately reflect real-world conditions, leading to potentially misleading conclusions about the intervention. One promising development in addressing certain limitations, such as reaching the target group, is the introduction of social media-based Theatre Testing.

## Introduction

Widely used in marketing research and advertising, Theatre Testing involves presenting materials to an audience in a controlled setting to gather feedback and insights. It provides companies with actionable insights to refine advertising campaigns and enhance marketing efforts. It has been a common practice for decades (Ostlund & Clancy, 1982), with early examples including showing a short television programme containing a series of rough-produced commercials to test the messaging with the audience (Freimuth & Greenberg, 1986). The application of the method to health communication feels natural; to maximise the effect of a health communication campaign, it is important to gain insight into public reactions. Accordingly, Theatre Testing features in health communication guidelines (National Institutes of Health, 2002). In recent years, there has been a broader application of Theatre Testing, including in assessing interventions prior to actual implementation. This has largely been prompted by the inclusion of the method in the ADAPT-ITT model (MacEntee et al., 2022; Wingood & DiClemente, 2008), a prescriptive eight-step approach to adapting evidence-based HIV interventions in which Theatre Testing features in step three, although examples extend beyond HIV intervention to other somatic (Pekmezaris et al., 2020) and mental health (Bitew et al., 2022) conditions as well as social interventions (Gupta et al., 2023).

While the application of Theatre Testing in marketing and advertising is long-standing, its increasing application in prevention science raises several critical questions. This article describes the Theatre Testing process, debates the merits and drawbacks of the method, exemplifies the pros and cons by discussing the method in relation to a community-based gender-based violence (GBV) intervention, and presents the concept of social-media-based Theatre Testing.

## Conducting Theatre Testing: A Step-by-Step Guide

**Fig. 1:**
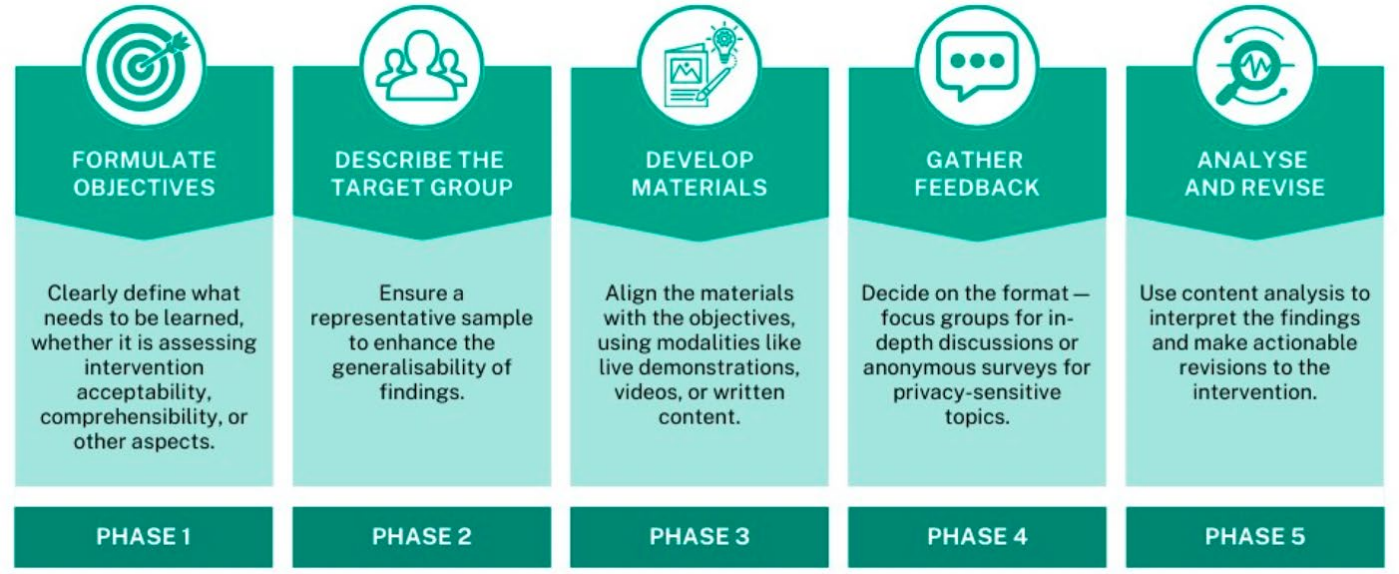
Step-by-step guide to Theatre Testing

Figure 1 provides an overview of the Theatre Testing process, as informed by the National Institutes of Health (2002). It is essential to clearly define the goals of the testing, such as assessing the comprehensibility or acceptability of a specific intervention or informing decisions related to intervention design. There may be alternative intervention design options to test or elements of the intervention that are undecided. Various modalities can be employed in Theatre Testing, including live demonstrations, video vignettes, visual aids, and written text. The materials used should align with the objectives of the testing. Additionally, the format for gathering feedback needs careful consideration. It is dependent both on the research question and the intervention format. Focus Group Discussions (FGDs) are beneficial for exploring complex or sensitive aspects of the intervention in detail. Sharing experiences and opinions in a group setting allows for rich and nuanced insights. FGDs are also efficient for data collection, as they capture multiple perspectives within a single session. However, it may sometimes be necessary to protect participants’ privacy and confidentiality; in such cases, an anonymous survey can be a viable option. Anonymous surveys allow participants to provide honest responses, especially on sensitive or personal topics, without fear of judgment or disclosure of their identity. This method can reach a broader and more diverse audience, including individuals who may hesitate to participate in FGDs due to privacy concerns. Furthermore, FGDs might inadvertently inflate the perceived acceptability of the intervention because of participant selection bias. Surveys offer a standardised method for data collection. Maintaining consistency in survey administration helps reduce potential bias and variability in the data collection process.

## The Case for Theatre Testing

It is important to ascertain how the target group responds to an intervention, and whether they consider it acceptable as this impacts engagement, adherence, and attendance (Moore et al., 2015). The intervention must be culturally appropriate, relevant to the target group, and satisfy personal preferences to be deemed acceptable. By systematically collecting and analysing data on target group responses to interventions, we can gain insights into the implementation process, identify areas of concern, and make recommendations to refine or adapt the intervention to better meet the needs of participants and achieve the intended outcomes (Moore et al., 2015).

Advocates of Theatre Testing argue that simulating interventions before implementation offers valuable insights into participant responses, which can enhance the efficacy of these interventions. The process can provide insights into challenges that may arise during the delivery of interventions. By identifying implementation challenges early, researchers can develop strategies to address them, ultimately improving the effectiveness and sustainability of the intervention. Understanding how interventions are implemented in real-world settings is essential for scaling up successful programs and disseminating them to broader populations. Theatre Testing also allows for the adaptation of interventions to better fit the needs and preferences of new target populations and the various contexts in which they may receive the intervention.

## Limitations of Theatre Testing

It is important to remember that Theatre Testing cannot guarantee positive participant responses in actual implementation. It is a pre-testing method to help refine interventions, rather than predict successful implementation. Whilst quantitative methods can be applied within Theatre Testing, one must resist seeking statistical precision. Instead, the results from Theatre Testing should be used to inform intervention refinement. The findings are not a substitute for experienced judgement, and intervention developers need to maintain the theoretical underpinnings of the intervention whilst taking on board insights from Theatre Testing. Like with all research, there is a risk of bias when performing Theatre Testing. There is a high risk of selection bias, in that those taking part in the testing do not represent the intervention target population. Researcher positionality also holds relevance for Theatre Testing; the social, cultural and political profile of the researcher can shape the construction and interpretation of the Theatre Testing process.

## Motivating Example

Table 1 outlines the details of a proposed GBV intervention to be implemented as part of a civic orientation course delivered at the Swedish ‘open preschool’. While the intervention has been co-designed with refugee women with lived experience of GBV (Pérez-Aronsson et al., 2024), several implementation questions remain which could be addressed using Theatre Testing. It is important to note that while Theatre Testing offers a way to engage the public in intervention development, those who take part are research participants rather than public contributors. That said, Theatre Testing can be conducted in a participatory manner in which public contributors are involved in the planning, conduct and interpretation of the Theatre Testing.

**Table 1:** Overview of the motivating example, informed by the template for intervention description and replication (TIDieR) checklist, a guideline developed to help improve completeness in the reporting of interventions in research studies (Hoffmann et al., 2014)

Name	A community-based intervention for promoting GBV help-seeking
Why	The purpose of the intervention is to create safe spaces for refugees in Sweden to acquire information about GBV, related services, rights and laws. Theorised outcomes include peer support and empowerment, as well as an increased number of refugees seeking help from GBV-relevant services. The proposed long-term impact is increased safety and well-being among refugees.
Materials	Picture vignettes, short animated movies, and discussion guides
Procedures	The intervention promotes discussion regarding GBV and available services. Connections are made with local services that use the course as a platform for outreach.
Provider	Open Preschool educators
How	Group-based, integrated into an existing civic orientation course
Where	The Swedish Open Preschool, a free-of-charge meeting place for parents and children aged 0 to 6 years staffed by trained educators who arrange activities for the children and offer parents advice and support.

The proposed GBV help-seeing intervention is to be integrated into an existing civic orientation course. It is unclear to the co-design team whether or not to explicitly label the intervention as targeting GBV. On one hand, explicitly labelling the intervention could aid reach; those experiencing GBV would perhaps be more likely to attend as it would be clear the intervention holds relevance for them. On the other hand, there is a heightened risk to participant safety. If GBV perpetrators become aware of the nature of the intervention they may prevent attendance and/or ongoing GBV could be aggravated. Thus, Theatre Testing can be used to determine– from the target group’s perspective– if the nature of the intervention content should be made explicit and, if so, the phrasing and terminology that could be used to do so. A further objective for the Theatre Testing of the GBV help-seeking intervention could be to test variations of the picture vignettes and/or animated videos, as well as discussion guide phrasings and choices of services to involve in the outreach activity.

In order to ask the target group whether or not to explicitly label the GBV help-seeking intervention and, if so, generate ideas for phrasing, it is important that the content and purpose of the intervention are clearly conveyed. This could, for instance, be achieved through an infographic showing the intervention content accompanied by video vignettes to demonstrate the intervention delivery format. Picture vignettes and animated videos could be shared directly. Discussion topic phrasings could be shared in written, audio or video format. Links to service websites could be shared to enable participant interrogation of the applicability and acceptability of their involvement.

When considering an intervention to promote help-seeking among refugee victim-survivors of GBV, the need to physically convene to take part in a Theatre Testing screening poses issues. The intervention itself requires physical attendance, yet a fundamental objective of Theatre Testing would be how to engage potential participants in a safe and acceptable way. Ironically, Theatre Testing carries the same risk of implementation failure as the intervention itself in that few people may attend and those who do may not represent the target group. Physical attendance raises concerns regarding participant privacy; it is challenging to uphold participant confidentiality and anonymity when data collection takes place at a public event. There could be a risk of harm to attendees if GBV perpetrators became aware of their interest in the intervention. We must also be sensitive to the potential for stigmatisation and discrimination among those who attend. Individuals who have been affected by GBV can face social devaluation, discrimination, and marginalisation (Murvartian et al., 2023, 2024). Moreover, the situational vulnerability of GBV victim-survivors may affect the power balance with researchers. Power differentials between the researchers and attendees could influence the dynamics of informed consent, voluntary participation, and the ability of participants to freely express their opinions or concerns. For a participatory project like ours, in which refugee victim-survivors of GBV collaborate with us in designing, conducting and interpreting the research, these risks extend to our team members; attendance at a physical Theatre Testing could pose a risk of harm or discomfort to our public contributors.

## A Role for Social Media-Based Theatre Testing?

Remote Theatre Testing can be adopted to test implementation without physically convening participants. Whilst remote participation has previously been adopted, this has been via video-conference interviewing which maintains the need for scheduled contact with researchers (Gupta et al., 2023). We instead suggest harnessing the extensive reach and asynchronicity of social media to conduct remote Theatre Testing. There is also greater potential for anonymity with social media contact. By enabling anonymity, the process can support thinking around how to safely, and meaningfully, convene at-risk groups for the intervention itself. Of course, anonymity comes with the methodological compromise of not knowing who is taking part and whether they meet eligibility criteria. Self-disclosure of key eligibility characteristics can be built into the process to somewhat mitigate against this.

The fundamental process of Theatre Testing remains unchanged (Fig. 1). For Phase 2, attention needs to be given to the selection of appropriate social media channels. We advocate for a participatory approach, in which public contributors from the target group disseminate the content. This aids the identification of suitable social media channels, which might be undiscoverable by researchers, facilitates a genuine connection with the target group and, in our case, supports dissemination in various languages relevant to the target group. A further potential benefit of the social media approach is the ability to protect the identity of public contributors. Artificial social media accounts could be created for Theatre Testing. While this somewhat compromises the ‘genuine’ connection with the target group, it does mean that public contributors who are interested in undertaking data collection but might be put at risk or discomfort by publicly assisting with the project can contribute to the process, as they could do so without needing to reveal their identity and compromise their safety. The artificial accounts could mimic the identities of the public contributors to still foster a connection with the target group.

For Phases 3 and 4, attention needs to be given to scope and format, which will be somewhat dependent on the chosen social media platforms. With average social media videos lasting under 30 s, a high level of prioritisation regarding content will be needed. It may be the case that a social media campaign is required, in which various intervention aspects are tested sequentially over a period of time. Data capture methods also need consideration. Web-based surveys are commonplace, and survey links can easily be included in social media posts. Survey brevity is key, given the fast pace of social media interactions. Anonymity will need to be emphasised to promote responses. One may also want to consider seeking ethical clearance to analyse comments left on social media posts, as comments are natural format of opinion exchange on social media. Whilst this form of data foregoes the structure awarded by a survey, it can be considered as an additional source of information to include in the analysis.

Although presented as a solution to some existing Theatre Testing issues, this social media approach comes with its own limitations. The scope for data capture is somewhat limited negating the possibility of in-depth research. To overcome this, researchers could use a combination of social-media-based and in-person Theatre Testing. Another pressing issue is the impact of social media. While the evidence is inconsistent, it appears as though social media use can adversely affect well-being (Valkenburg, 2022), which brings into question whether we as researchers should encourage social media consumption. Yet, the intention would be to reach already active users. When considering the impact of social media, one must consider the context of engagement; contributing knowledge to intervention development can empower individuals, fostering a sense of purpose and creating the opportunity to shape solutions that can benefit themselves and others.

## Conclusion

While Theatre Testing offers a promising tool for prevention scientists, it is crucial to remain cognisant of its limitations. Methodological advancements, particularly those involving social media, hold the potential to address some of these concerns but come with their own limitations. As the debate continues, it is essential to critically appraise evidence from forthcoming publications that utilise this method to ensure that the insights gained are valid and applicable to real-world settings.
